# Comparison of self-reported and performance-based measures of functional ability in elderly patients in an emergency department: implications for selection of clinical outcome measures

**DOI:** 10.1186/s12877-016-0376-1

**Published:** 2016-11-29

**Authors:** Louise M. Nielsen, Hans Kirkegaard, Lisa G. Østergaard, Karina Bovbjerg, Kasper Breinholt, Thomas Maribo

**Affiliations:** 1 Department of Physiotherapy and Occupational Therapy, Aarhus University Hospital, Aarhus C, Denmark; 2 School of Occupational Therapy at VIA University College, Aarhus N, Denmark; 3 Research Center for Emergency Medicine, Aarhus University Hospital, Aarhus C, Denmark; 4 MarselisborgCentret, DEFACTUM, Central Denmark Region, Department of Public Health, Aarhus University, Aarhus C, Denmark

**Keywords:** Assessment, Performance-based, Self-report, Daily activities, Acute care, Functional ability, Disability

## Abstract

**Background:**

Assessment of functional ability in elderly patients is often based on self-reported rather than performance-based measures. This study aims to compare self-reported and performance-based measures of functional ability in a population of elderly patients at an emergency department (ED).

**Methods:**

Participants were 61 patients aged 65 years and above admitted to an ED. The self-reported measure used was the Barthel-20; the performance-based measures were Timed Up and Go (TUG); 30s-Chair Stand Test (30s-CST) and Assessment of Motor and Process Skills (AMPS) with the two scales; motor and process. Correlation analyses were conducted to examine the relationships between the self-reported and performance-based measures of functional ability.

**Results:**

The correlation between the Barthel-20 and the TUG was moderate (*r* = −0.64). The correlation between the Barthel-20 and the AMPS motor was also moderate (*r* = 0.53). The correlation between the Barthel-20 and the 30s-CST was fair (*r* = 0.45). The correlation between Barthel-20 and the AMPS process was non-significant. The results were affected by high ceiling effect (Barthel-20).

**Conclusion:**

Self-reported and performance-based measures seem to assess different aspects of functional ability. Thus, the two methods provide different information, and this highlight the importance of supplementing self-reported measures with performance-based measures when assessing functional ability in elderly patients.

**Electronic supplementary material:**

The online version of this article (doi:10.1186/s12877-016-0376-1) contains supplementary material, which is available to authorized users.

## Background

Elderly patients often experience limitations in their functional ability related to daily activities and mobilization [1–4]. Limited functional ability in elderly is associated with increased risk of readmission and may be a predictor of prolonged hospitalisation and increased mortality [5–7]. According to a systematic review by Wales et al., assessment of functional ability is the first step in identifying rehabilitation needs in elderly patients and to determine effectiveness of treatment [8]. Assessment of functional ability is also an important element in the multidimensional Comprehensive Geriatric Assessment (CGA) approach aiming at providing care and treatment for geriatric patients. The CGA assesses the patient's level of independence in performing daily activities using measures as Barthel Index, the Katz Index of independence in Daily Activities and the Function Activity Questionnaire [9]. However, there is currently no consensus on the use of a “gold-standard” for assessment of functional ability in elderly [10–13].

There are different approaches to assessing functional ability and several outcome measures related to different aspects of functional ability are used [10, 11]. Self-reported measures are frequently used to assess functional ability in elderly patients [10, 12, 14]. Self-reporting may be less time-consuming than performance-based assessment but the two approaches vary considerably [14]. Two studies have compared self-reported and performance-based assessment of functional ability in elderly patients and they found little overlap between the two methods [15, 16]. A study of Roedersheimer et al. [17], found discrepancies between self-reported ability and performance-based ability in simple mobility tasks. In general, both approaches to data collection have advantages as well as limitations. One important feature of the self-reported approach is that it represents the patient perspective. However, the use of self-reported data in emergency departments poses several limitations. Patients hospitalised for medical problems may experience a sudden, but unrecognised decline in functional ability and their self-reporting of current functional ability may consequently not be accurate. Furthermore, self-reporting in elderly patients can be problematic due to cognitive impairment or affective responses to acute illness [16]. On the other hand, performance-based measures can be used to evaluate discrete and specific components of the performance on specific tasks, including how the task was approached. This can point to specific disabilities which can be targeted during treatment and rehabilitation [18–20]. Although performance-based measures seems to have some advantages, evidence indicate they are not routinely performed in ED [12, 21].

The aim of the present study was to compare self-reported and performance-based measures of functional ability in elderly patients at an ED.

## Methods

### Design and setting

The study used a cross sectional design and was conducted at a university hospital in Denmark.

### Study participants

Patients were recruited from the ED at a university hospital. Patients aged 65 years or older with planned discharge directly from the ED, who were able to sit on a chair were included. Exclusion criteria: Orthopaedic patients, patients admitted from a nursing home, patients requiring palliative care, patients not speaking Danish and patients unable to follow instructions due to cognitive impairment. Patients were tested after acute medical treatment and close to discharge. The assessments were performed for the purpose of this study and were thus not a part of the daily routine.

Written informed consent was obtained to perform the assessments and use the data for the purpose of this study. The study was approved by the Central Denmark Region Committees on Biomedical and Research Ethics (J. nr.1-10-72-108-14), and by the Danish Data Protection Agency (J.nr. 2012-41-0763).

### Outcome measures

#### Self-reported measure

One of the most commonly used functional outcome measures in elderly patients both in research and in clinical settings is the Barthel Index [22–26]. The index measures a person’s level of independence in the performance of daily activities. It is an ordinal scale comprising ten activities including grooming, bathing, feeding, getting on and off the toilet, ascending and descending stairs, getting dressed bladder continence, bowel continence, walking, and transferring. Although it is widely used, a study by de Morton [27] found that the index was not unidimensional and that the scale consists of different constructs.

A widely adopted modification by Collin and Wade [26] uses a score range from 0 (high dependence on assistance) to 20 (independent of assistance). The Barthel-20 can be used as self-reporting, by proxy and as an observation-based measurement [28].

#### Performance-based measures

To identify performance-based outcome measures for functional ability in elderly patients, we searched the literature. Selection criteria were: Generic outcome measures validated for the elderly population and simple to administer without the use of special equipment.

Based on the literature, Timed Up and Go (TUG) and 30s-Chair Stand Test (30s-CST) were chosen to assess disability in relation to basic mobility [29, 30]. Both measures are widely used at medical and geriatric departments and they are validated and feasible for use in elderly hospitalised patients [31–33]. To get a broader perspective on functional ability [34], we supplemented measures of mobility with measures of quality in performance of daily activities. Here the Assessment of Motor and Process Skills (AMPS) is the only performance-based instrument that measures a person’s quality of performing daily activities [18, 19, 35].

The TUG test assesses basic mobility and reflects a person’s ability to get up from a chair, walk three metres and turn around. Wearing regular footwear and using his/her customary walking aid, participants were asked to complete the following as fast and safely as possible: Get up from an armchair (46 cm high), walk three metres (marked by tape), turn, return and sit down. Timing begins at the instruction “go” and stops when the person is seated. The faster a person is, the better and a score < 20 s reflects independence in basic transfers [29]. When possible, the best result in seconds of three attempts was recorded and used for analysis [29].

The 30s-CST assesses lower body strength and area of functional mobility. It uses a chair with a seat height of 43 cm and no arm rest. At the signal “go”, the participant rose to a full stand and was instructed to complete as many full stands as possible within the 30-s time limit [30].

The AMPS measures the quality of a person’s performance of daily activities in natural, task-relevant environments [18]. It is an observational assessment instrument used by occupational therapists (OT) with an AMPS license. The AMPS consists of two scales, one measuring motor skills and one measuring process skills. Computer scoring of the AMPS provides logit values from −4 to +4. AMPS indicate whether the patient is able to live independently in the community or whether minimal, moderate or maximal assistance is needed [36].

#### Data collection

Data on diagnosis and age was collected from the patient’s medical record. A research physiotherapist (PT) used the Barthel-20 as self-report during a face-to-face interview. The time frame used in the Barthel-20 was one week. Afterwards, the patient was tested using the TUG and the 30s-CST administered by a PT and AMPS administered by a OT. The Barthel-20 was always administered first so that the Barthel-20 score was not affected by the patient’s test performance. All tests were performed in accordance with a standard protocol. None of the performance-based measures was part of routinely praxis and the two OT’s was licensed to administer AMPS in relation to the study.

#### Hypothesis

The following a priori hypotheses tested were based on the assumption that measures that conceptually converge should be strongly correlated and measures with less in common should have a weaker correlation. As the measures represent different constructs of functional ability, we hypothesised the following:

- The correlation between Barthel-20 and TUG would be fair.
- The correlation between Barthel-20 and 30s-CST would be fair.
- The correlation between Barthel-20 and AMPS motor skills would be fair.
- The correlation between Barthel-20 and AMPS process skills would be fair.

### Data analyses

Descriptive statistics were used to report frequency and proportions for categorical variables, medians were reported with 5 and 95 percentiles for data on ordinal scales and means and standard deviations for continuous variables with normal distribution. Floor and ceiling effects were examined and described for Barthel-20, TUG and 30s-CST. Such effects occur if more than 15% of the patients achieve the lowest or highest possible score [37].

The relationship between self-reporting and performance-based measures was examined with Spearman’s correlations between TUG, 30s-CST, AMPS and Barthel-20, respectively. Coefficients were stated as poor (<0.25), fair (0.25-0.49), moderate (0.50-0.74) and excellent (≥0.75) [38]. Analyses were conducted using Stata-13.0.

## Results

**Fig. 1:**
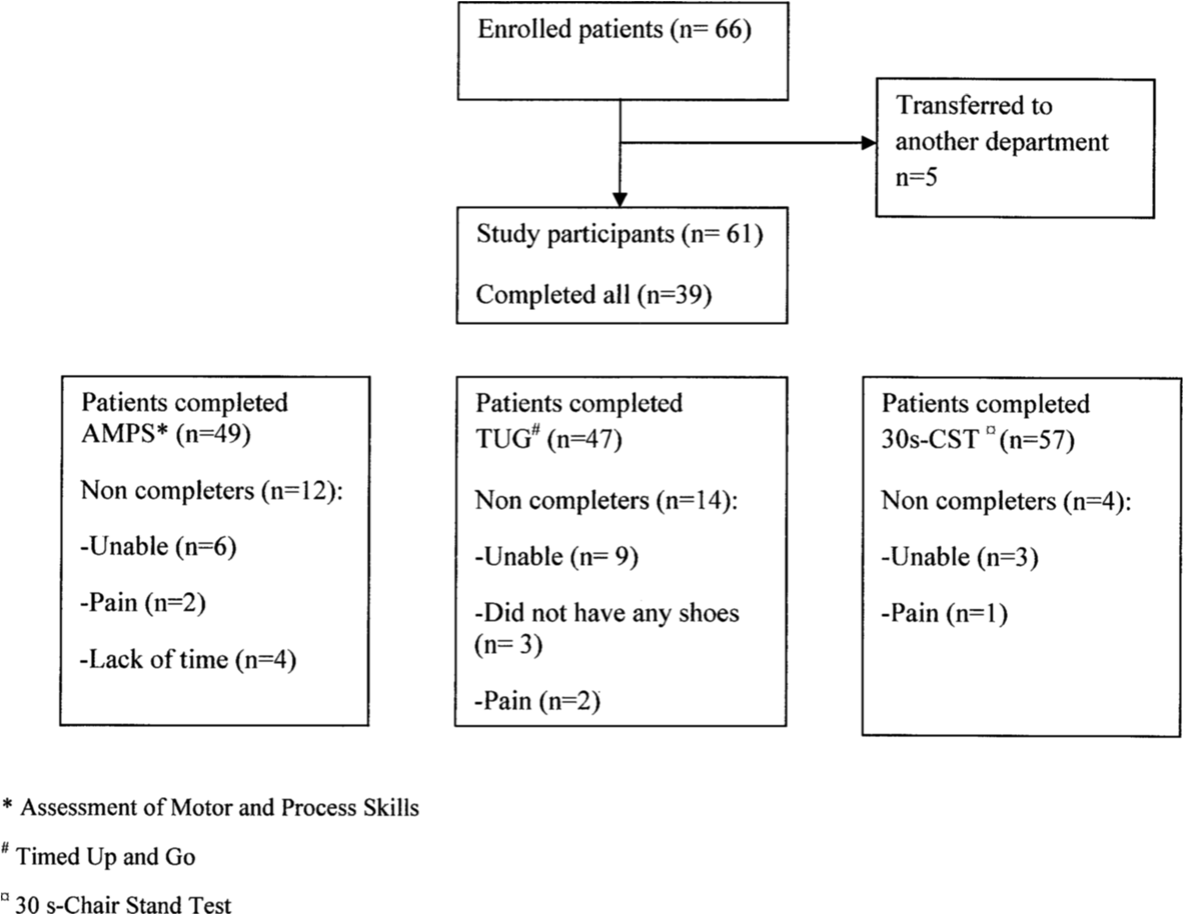
Flowchart of study participants

Sixty-six patients were enrolled in this study; five patients were transferred to another department instead of being discharged and were excluded. The final study population thus comprised 61 participants all completed Barthel-20; 77.1% completed the TUG, 80.3% the AMPS and 93.4% the 30s-CST. Reasons for not completing the tests are described in Fig. 1. There was no difference between completers and non-completers in relation to gender, age or Barthel-20 score (Fig. 1).

The mean age of participants was 80.4 years (SD 7.7) and 57% were women. A large proportion (20%) had no specific diagnosis (Table 1). The median scores were 19 (11–20) for the Bathel-20, 13.7 (6.4-39.6) for the TUG, 4 (0–14) for the 30s-CST, 0.97 (SD 0.76) for the AMPS motor skills and 0.73 (SD 0.67) for AMPS process skills (Table 1).

**Table 1:** Characteristics of study population (*n* = 61)

Characteristics		
Female, n (%)	35	(57%)
Age, mean (SD)	80.4	(7.7)
Primary diagnosis:		
Urinary tract disease, n (%)	8	(13%)
Endocrine disorders, n (%)	9	(15%)
Respiratory disease, n (%)	7	(11%)
Heart disease, n (%)	5	(8%)
Disease of the bones and muscles, n (%)	5	(8%)
Other conditions, n (%)	15	(26%)
Symptoms of conditions, n (%)	12	(20%)
Barthel-20 score, median (5/95 percentile)	19	(11–20)
Timed Up and Go ^a^, median (5/95 percentile)	14	(6–40)
30s. Chair-Stand Test ^b^, median (5/95 percentile)	4	(0–14)
AMPS motor^c^, mean (SD)	0.97	(0.76)
AMPS process^3^, mean (SD)	0.73	(0.67)

^a^ *n* = 47, ^b^ *n* = 57, ^c^ *n* = 49

**Fig. 2:**
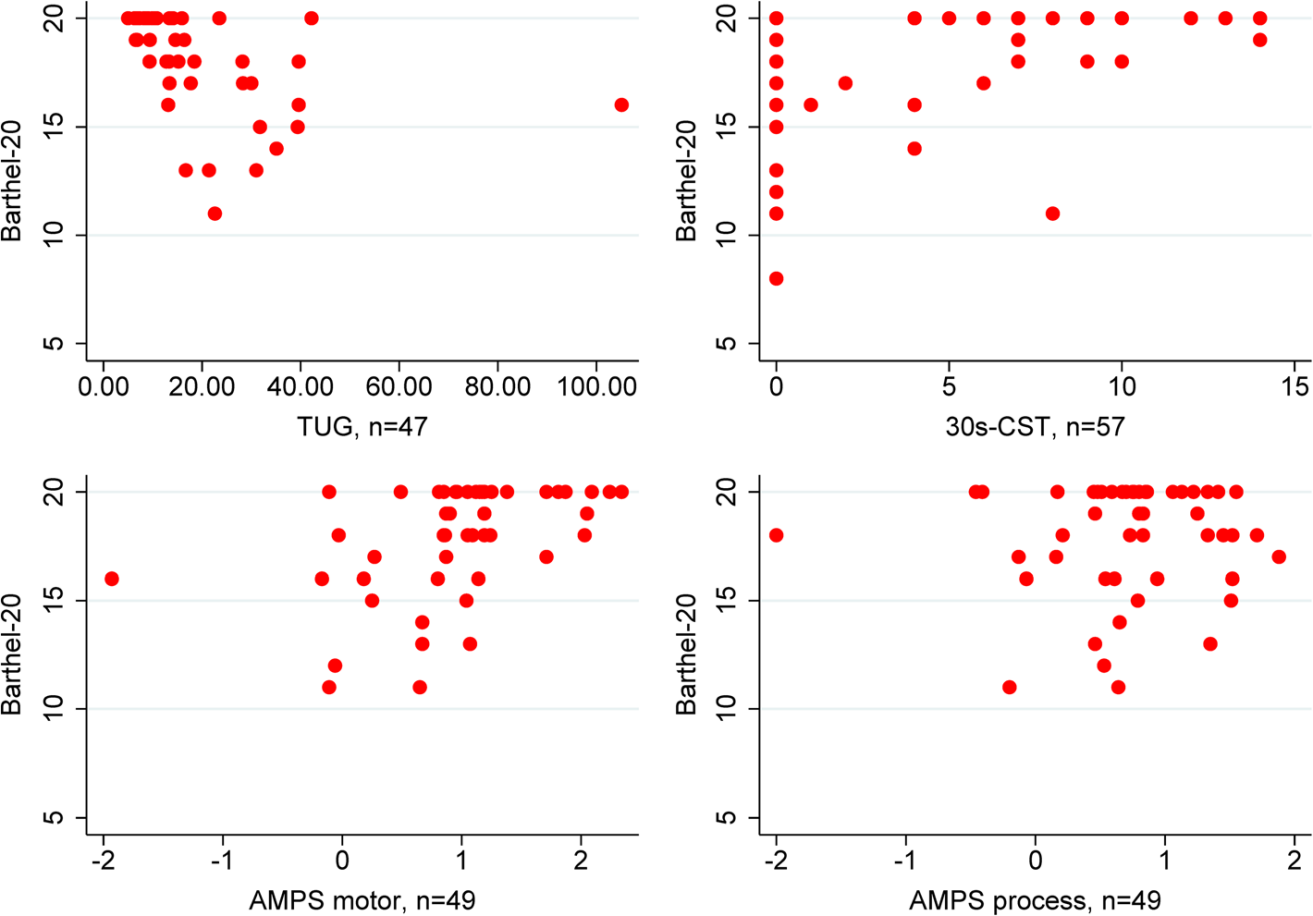
Plots of correlation between Barthel-20, Timed Up and Go, 30s. Chair-Stand Test and Assessment of Motor and Process Skills

As shown in Fig. 2, the Barthel-20 had a ceiling effect as 43% of the patients scored the highest possible score. Both TUG and 30s-CST had floor effect; 23% of the patients scored 0 in TUG and 44% of the patients scored 0 in 30s-CST.

The correlation between Barthel-20 (level of independence) and TUG (basic mobility) was moderate (*r* = −0.64). The correlation between Barthel-20 (level of independence) and AMPS motor skills (quality of performance of daily activities – motor skills) was moderate (*r* = 0.53), while the correlation between Barthel-20 (level of independence) and 30s-CST (lower body strength) was fair (*r* = 0.45). The correlation between Barthel-20 and AMPS process skills (quality of performance of daily activities – process skills) was poor (*r* = 0.06) (Table 2).

**Table 2:** Correlation between Barthel-20, Timed Up and Go, 30s. Chair-Stand Test and Assessment of Motor and Process Skills

	TUG^a^	30s-CST	AMPS Motor	AMPS Process
*Barthel-20*				
r (95%CI)	−0.64 (−0.79:-0.44)	0.45 (0.22:0.64)	0.53 (0.29:0.71)	0.06 (−0.23:0.33)
n	47	57	49	49
*Barthel-20 mobility*				
r (95%CI)	−0.66 (−0.79:-0.45)	0.38 (0.13:0.58)		
n	47	57		
*Barthel-20 activities* ^b^				
r (95%CI)			0.62 (0.41:0.77)	0.24 (−0.04:0.49)
n			49	49

^a^Correlations with the TUG test were expected to be negative as lower score in TUG reflects better outcome

^b^Items 1,3,4,5,8 in the Barthel-20 are related to daily activities

Barthel-20 comprises two different constructs: mobility and daily activities. Thus, we divided Barthel-20 into two sub-scores related to daily activities (items 1, 3, 4, 5 and 8) and mobility (items 2, 6 and 7) to test if the correlations became stronger. None of the correlations changed significantly (Table 2).

## Discussion

This study compared self-reported and performance-based measures of functional ability in elderly patients at an ED. The correlation between Barthel-20 and TUG and between Barthel-20 and AMPS motor skills was moderate (r: 0.50 - 0.74). It was hypothesised that the correlation would be only fair because the measurements represent different constructs. The correlation between Barthel-20 and 30s-CST was fair and the correlation between Barthel-20 and the AMPS process skills was poor. This may either indicate a difference between the different underlying constructs (mobility and performance of daily activities) of the Barthel-20 and the three performance-based measures or confirm our hypothesis that self-reporting compared with performance-based measurements provides distinct information about functional ability [15, 16].

The three performance- based measures revealed a higher prevalence of patients with functional limitations compared to the self-reported measure (Barthel-20). One explanation could be that the patients were not yet aware of their ability to mobilize or to perform daily activities due to their state of sudden acute illness and admission to hospital. Our results are in accordance with results from other studies. A study by Wæhrens et al. [39] found that measures of self-reported daily activities had limited correlation to observed performance of daily activities in a population of women with rheumatoid arthritis, knee osteoarthritis or fibromyalgia. Sager et al. [16] found significant differences between elderly patients’ self-reported performance of daily activities and performance-based assessments of daily activities at the time of discharge. The same tendency was reported by Roedersheimer et al. [17] who found a discrepancy between elderly patients’ self-reported ability to perform simple mobility tasks and results of their performance-based abilities. In the present study we compared measures of functional ability with different underlying constructs and our results are thus not directly comparable. We could have compared the Barthel-20 used as a self-reported measure with the Barthel-20 used as a performance-based measure, but to the best of our knowledge assessment of functional ability encompasses more than level of independence in performing daily activities.

The difference between self-reported and performance-based measures could have implications for the patient’s discharge and further rehabilitation process. Self-reported measures of functional ability may be inadequate in the planning of rehabilitation, especially as some patients are unable to report their performance realistic and accurately.

Assessing functional ability during hospitalisation may prevent the patient from undergoing further testing and the assessments can be used by other professionals during the course of rehabilitation, thus potentially improving the quality of overall pathway. Investing time and resources in obtaining comprehensive knowledge of older patients is an important part of the Comprehensive Geriatric Assessment (CGA) [40, 41]. Including performance-based measures in this assessment may not only ensure that patients receive the right care, training, and rehabilitation; such efforts may also be instrumental in reducing mortality and readmission rates seen among elderly disabled patients [2, 6].

The high ceiling effect of the Barthel-20 in our study indicates the challenges of using this test. Moreover, it also suggests that self-reported measurements of functional ability in general may be problematic in elderly patients in an acute care setting [14, 15].

Both the TUG and the 30s-CST showed floor effects. This indicates that the tests are not sufficiently sensitive for use in all older patients [37]. A large part of patients (44%) were not able to follow the protocol of the 30s-CST and rise without the use of armrest. Perhaps we should have categorized these data differently to get a broader picture of the patients’ physical performance. A study by Bodilsen et al., [42] describe three categories 1) ability to rise without using the armrest, 2) ability to rise using the armrest and 3) inability to rise independently from the chair. In our study, 17 patients completed the TUG, but scored 0 in the 30s-CST. This indicates that at least these 17 patients would score 2 using the scale presented by Bodilsen [42].

Our study is limited by a relatively small sample size. Nevertheless, an important strength of the study is that several measures are examined in the same sample, which mitigates the potential risk of comparing measures across different samples. This strengthens our result that self-reported and performance-based measures seem to assess different aspects of functional ability. About 20% of the study population was unable to complete either the TUG or the AMPS. This suggests that the measures may not be suitable in the entire population, or perhaps that the use of all three measures was too cumbersome for some of the patients in the ED. The use of AMPS can reveal some limitations, as the measure requires license of the OT.

Another limitation is that we included only patients able to sit on a chair and who were able to follow instructions. The findings may therefore not be generalizable to all elderly patients at an ED.

Our findings add to the growing evidence that self-report and performance-based measures of functional ability provide distinct and different information. These differences are relevant to both clinicians and researchers. It should be recognized that the Barthel-20 does not provide nearly as broad and compressive a view of functional ability as the three performance-based measures. The underlying construct of the Barthel-20 is different from the constructs of the performance-based measures. Thus, direct comparisons might be challenging. However, both self-reported and performance-based measures describe aspects of functional ability and both are advocated outcomes of this domain within clinical research [16, 39]. A priority for further research should be to examine if or how the use of both performance-based and self-reported measures of functional ability leads to improved discharge planning, rehabilitation, and thus better patient outcome.

## Conclusion

In conclusion we found that patients reported higher functional ability than observed by using the performance-based measures. This indicates that the two methods provide different information about functional ability. Thus, it is important to supplement self-reported measures with performance-based measures as both methods provide important and complementary information about the elderly patients’ functional ability.

## Additional file

Additional file 1: Data used for analysis. (XLS 33 kb)

## Abbreviations

30s-CST: 30s- chair stand test
AMPS: Assessment of motor and process skills
CGA: Comprehensive geriatric assessment
ED: Emergency Department
TUG: Timed up and go
